# Enhancing bloodstream infection diagnostics: a novel filtration and targeted next-generation sequencing approach for precise pathogen identification

**DOI:** 10.3389/fmicb.2025.1538265

**Published:** 2025-03-20

**Authors:** Ting-Syuan Lin, ZiHao Zhu, XiaoHong Lin, Hsi-Yuan Huang, Li-Ping Li, Jing Li, Jie Ni, PeiZhi Li, LanChun Chen, WeiXin Tang, HuiXin Liu, XiaoLong Se, MingFei Xie, Canling Long, Chih-Min Chiu, Szu-Han Fang, JiaMing Zhao, Yang-Chi-Dung Lin, XueTao Yu, Hsien-Da Huang

**Affiliations:** ^1^School of Medicine, The Chinese University of Hong Kong, Shenzhen, Guangdong, China; ^2^Warshel Institute for Computational Biology, School of Medicine, The Chinese University of Hong Kong, Shenzhen, Guangdong, China; ^3^Guangdong Provincial Key Laboratory of Digital Biology and Drug Development, The Chinese University of Hong Kong, Shenzhen, Guangdong, China; ^4^Department of Critical Care Medicine, The Second Affiliated Hospital, School of Medicine, The Chinese University of Hong Kong, Shenzhen & Longgang District People’s Hospital of Shenzhen, Shenzhen, Guangdong, China; ^5^Shanya life-tech Co. Ltd., Guangzhou, Guangdong, China; ^6^Central Laboratory, The Second Affiliated Hospital, The Chinese University of Hong Kong, Shenzhen & Longgang District People’s Hospital of Shenzhen, Shenzhen, China; ^7^Health SwifTech Co. Ltd., Shenzhen, Guangdong, China; ^8^Department of Endocrinology, Key Laboratory of Endocrinology of National Health Commission, Peking Union Medical College Hospital, Chinese Academy of Medical Sciences & Peking Union Medical College, Beijing, China

**Keywords:** bloodstream infections, pathogen, whole blood, filtration membrane, targeted next-generation sequencing

## Abstract

Bloodstream infections (BSIs) pose a significant diagnostic challenge, largely due to the limitations of traditional methods such as blood cultures. These methods often yield low positive rates, have lengthy processing times that delay treatment, and are limited in detecting only a narrow range of pathogens. Such delays and inaccuracies can critically impede timely clinical interventions, potentially compromising patient outcomes. Next-generation sequencing (NGS) is a powerful tool for rapid, precise pathogen identification. While metagenomic NGS (mNGS) offers broad pathogen coverage, it is often costly and complex. Targeted NGS (tNGS), however, focuses on key regions of clinically relevant pathogens, reducing costs and simplifying workflows while maintaining high sensitivity, making it more practical for routine diagnostics. In this study, we introduce a novel approach combining a human cell-specific filtration membrane with a multiplex tNGS panel to overcome these challenges. The filtration membrane, designed with surface charge properties to be electrostatically attractive to leukocytes for the selective capture of specific cells, demonstrated high efficiency in removing host cells and nucleic acids, achieving over a 98% reduction in host DNA and thereby minimizing background interference in pathogen detection. Additionally, we developed an effective multiplex tNGS panel targeting over 330 clinically relevant pathogens and verified its consistency with mNGS and blood culture results, demonstrating a significant improvement in detection sensitivity. By integrating these two methods, we achieved a synergistic enhancement in diagnostic capability, boosting pathogen reads by 6- to 8-fold, which enabled reliable identification even in cases of low-abundance pathogens. This approach provides faster, more accurate, and more sensitive detection of BSIs, enabling earlier identification of infections. This facilitates timely and targeted treatment, ultimately improving patient outcomes in critical care settings. Given the unique properties of the filtration membrane and the strengths of the tNGS panel, this approach shows promising applications in prenatal and genetic health support, as well as in advancing early cancer screening strategies.

## 1 Introduction

Identifying unknown pathogen infections is a critical challenge in clinical diagnostics due to the lack of specific symptoms and the limitations of conventional methods, such as blood cultures, which are often slow, prone to contamination, and may yield false-negative results (Gupta et al., 2023). These infections can lead to severe systemic responses, including systemic inflammation and organ failure, particularly in vulnerable populations like infants, the elderly, and immunocompromised individuals (Chen et al., 2023). Bacterial infections, such as those causing pneumonia and kidney and abdominal infections, are among the most common triggers of severe systemic infections (Channon-Wells et al., 2023). However, viral agents (e.g., influenza, COVID-19) and, less frequently, fungi can also be culprits (Barbe et al., 2022). Early identification and targeted treatment are essential to mitigate the risk of severe complications, with preventative measures like vaccination playing a supportive role (Monnet et al., 2023; Huabbangyang et al., 2023).

Currently, there is no single definitive test for rapidly diagnosing these infections. While tests like complete blood counts, lactate levels, and blood cultures offer supportive but inconclusive evidence. Blood cultures—the current gold standard—require several days to yield results, delaying critical interventions (Liu and Mu, 2023; Han et al., 2019). The delay in diagnosis hinders timely and targeted treatment and increases the risk for patients who may not exhibit severe symptoms until the infection has progressed (Yan et al., 2022). This underscores an urgent need for more accurate and efficient diagnostic methods to identify pathogens quickly and enable timely, appropriate clinical responses (Li et al., 2023).

Advances in next-generation sequencing (NGS) technologies and bioinformatics tools have significantly accelerated the development of human pathogen identification research. Among the diagnostic technologies addressing this need is metagenomic next-generation sequencing (mNGS). A key feature of mNGS is its broad-range pathogen detection capability, enabling rapid and unbiased testing (Li et al., 2022). However, several technical factors can influence its detection range. These include the efficiency of pathogen nucleic acid extraction, the loss of pathogens due to improper removal of human nucleic acids, the accuracy and specificity of bioinformatic analyses, and the comprehensiveness and quality of pathogen data within reference databases (Xu et al., 2023; Yang et al., 2023; Zhao et al., 2023). Targeted next-generation sequencing (tNGS) addresses some of these limitations by enriching pathogen-specific sequences prior to library preparation and sequencing, making it a promising diagnostic approach (Deng et al., 2023). Various enrichment methods, such as CRISPR-Cas9 (Malekshoar et al., 2023), probe hybridization (Wang et al., 2021), and PCR amplification (Li et al., 2025; Cai et al., 2024), can be employed. Compared to metagenomic approaches, tNGS mitigates the “needle in a haystack” challenge when detecting low-abundance pathogens in samples with high background noise (Ballester et al., 2016; Xia et al., 2023). However, enrichment procedures, such as multiplex PCR for specific genes, can introduce target bias (Gao et al., 2021; Maccio et al., 2021; Visser et al., 2016; Ding et al., 2012). Moreover, while rapid diagnostic techniques are effective, they are often expensive and susceptible to false positives (Wang et al., 2023). This is primarily due to the high background of human DNA in samples, which can obscure or mimic signals from low-abundance pathogens, leading to inaccurate identifications (Perlejewski et al., 2020). Substantial human DNA content may overwhelm signals from less abundant species, underscoring the necessity of host DNA depletion techniques prior to sequencing (Sun et al., 2024). Several host DNA depletion strategies have been developed to enhance microbial detection in clinical samples. One approach involves microbial DNA enrichment, as demonstrated by Hasan et al. (2016), who showed that commercial kits such as the NEBNext Microbiome DNA Enrichment Kit and the MolYsis Basic Kit can significantly improve the microbial-to-human DNA ratio in samples with low pathogen abundance, achieving up to 9,580-fold enrichment. However, these methods vary in efficiency; while the MolYsis kit provides greater enrichment, it may introduce biases and increase processing complexity, highlighting the need for alternative depletion strategies that optimize efficiency, cost, and usability in next-generation sequencing (NGS)-based pathogen detection. Ji et al. (2020) demonstrated that saponin-mediated selective lysis and centrifugation-based removal of human cells can improve microbial detection, with centrifugation showing enhanced sensitivity in diagnosing both tuberculous and cryptococcal meningitis. Despite their potential, these methods may have variable effectiveness across different sample types, underscoring the need for further refinement of host DNA depletion strategies to maximize sensitivity and specificity in clinical metagenomic applications. These strategies are essential for improving the sensitivity and specificity of pathogen detection in clinical samples.

Differential centrifugation, commonly used for blood component separation, enables the isolation of red blood cells, white blood cells, and platelets by adjusting speed and duration. This approach is effective for reducing host DNA content by preliminarily separating white blood cells. However, additional methods are often necessary to achieve greater purity or specificity. Various techniques have been explored for bacterial separation, including density gradient centrifugation (Sun and Sethu, 2018), inertial and elastoinertial microfluidics (Urbansky et al., 2017), surface acoustic waves (SAW) (Qi et al., 2023), dielectrophoresis (DEP) (Moore et al., 2023), and magnetic bead-based separation (Pearlman et al., 2020). Nevertheless, these methods present limitations such as low throughput, low separation efficiency, reliance on high bacterial concentrations, or selectivity for specific bacterial strains.

**Figure 1:**
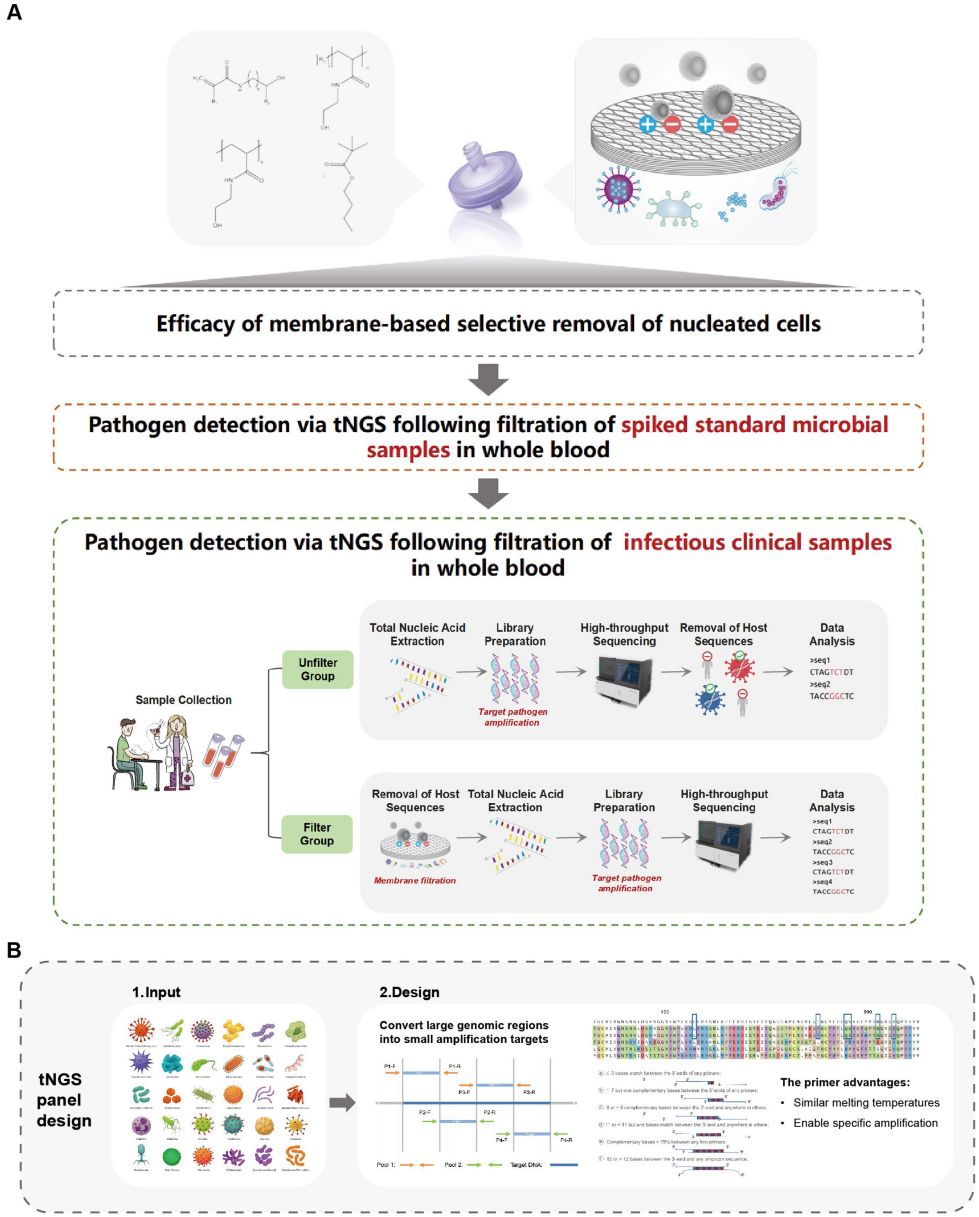
Research framework overview. **(A)** Efficacy of membrane-based selective removal of nucleated cells. **(B)** Schematic diagram of tNGS panel design.

In this study, we introduce a novel human cell-specific filtration membrane designed to pre-treat clinical samples effectively. During sample transportation, mechanical vibrations or external factors can cause white blood cells in blood samples to rupture, releasing host DNA and increasing the abundance of background sequences in the sample. This filtration device addresses this critical issue by reducing the host DNA content, thereby enhancing the detection of low-abundance pathogen sequences during tNGS analysis. Furthermore, we have developed a comprehensive tNGS panel that covers over 330 clinically common pathogens, representing over 95% of known infection types, including *Staphylococcus aureus*, *Klebsiella pneumoniae*, *Candida albicans*, and Influenza virus, among others. Our method not only reduces human DNA background but also increases pathogens concentration in the blood and streamlines the pre-treatment process of samples. The introduction of this innovative filtration membrane, alongside the extensive tNGS panel, has the potential to revolutionize clinical diagnostics by offering a more efficient, precise, and rapid method for detecting pathogens, ultimately enhancing patient care outcomes (Figure 1A).

## 2 Materials and methods

### 2.1 Human cell-specific filtration membrane

To address the issue of high background noise, we developed a method and apparatus to enrich and detect microbes in biological samples. In this method, the biological samples are filtered through a substrate designed to capture nucleated cells (CN113444767A), integrated in a human cell-specific filtration membrane (Health SwifTech, China). This substrate exhibits high specificity for capturing or separating nucleated cells. The distinctive feature of the substrate capable of capturing nucleated cells lies in its composition, which includes materials such as leukosorb membranes, triacetate cellulose, acetate cellulose, glass cellulose, quartz cellulose, nitrate cellulose, regenerated cellulose, or nylon-based substrates (Supplementary Figure S1). Microorganisms, including bacteria, mycoplasma, fungi, viruses, and spores, can pass through or flow past the nucleated cell-capturing substrate into the filtrate. This process enriches the microbial content of the biological samples during the reduction of nucleated cells, such as leukocytes, thereby minimizing the interference in pathogen detection caused by these cells.

The filtration process was conducted through the following systematic steps. First, a syringe needle was carefully inserted into the patient’s vein at an appropriate angle to collect blood. Following blood collection, the needle was detached from the syringe and replaced with a filtration membrane, which was then connected to a needle below the membrane. A vacuum blood collection tube was attached to the needle-filter assembly, enabling filtration. During the filtration step, the negative pressure within the vacuum tube drove the blood through the integrated filtration membrane, completing the filtration process.

### 2.2 Targeted next-generation sequencing (tNGS) and analysis

#### 2.2.1 Genomic data integration and primer design

Genomic data were integrated from multiple databases [FDA-ARGOS (Sichtig et al., 2019), GenBank (Sayers et al., 2022), FungiDB (Stajich et al., 2012), RefSeq (Tatusova et al., 2015), RVDB (Goodacre et al., 2018), nr (Wilke et al., 2012), and CARD (Alcock et al., 2023)], and representative pathogen strains were selected based on temporal and regional epidemiological prevalence. High-quality reference genomes with deep sequencing coverage and comprehensive annotations were prioritized, while potential contaminants (e.g., engineered reagent strains or environmental microbes) were systematically excluded. Pathogen-specific tag sequences were extracted using in-house bioinformatics algorithms, followed by saturated primer design to ensure full sequence coverage for broad-spectrum detection. Primer parameters were optimized with a length of ~20 bp, melting temperature (Tm) of ~60°C, GC content of 30–65%, and strict control of 3′ end complementarity to avoid primer-dimer formation.

#### 2.2.2 Amplicon optimization and panel validation

The resulting amplicons were designed to meet downstream sequencing requirements, with lengths of 150–250 bp, GC content of 30–65%, and strict specificity to uniquely target pathogens while maintaining broad strain coverage. Panel performance was rigorously validated through limit of detection (LOD) analysis, reproducibility testing, interference resistance assessment, and consistency verification with clinical samples.

#### 2.2.3 Nucleic acid extraction

Total DNA and RNA were co-extracted from the samples using the AccuGen Nucleic Acid Extraction (DNA/RNA) Kit (Health SwifTech, China) following the manufacturer’s protocol. The extracted nucleic acids were eluted in nuclease-free water and the concentration was quantified using the Qubit dsDNA HS Assay Kit (Thermo Fisher Scientific, USA), while RNA integrity was assessed using the Agilent 2100 Bioanalyzer (Agilent Technologies, USA).

#### 2.2.4 Targeted pathogen enrichment using multiplex PCR

To enhance the detection of pathogenic nucleic acids, targeted enrichment was conducted using a multiplex PCR-based approach with pathogen-specific primers (Figure 1B). The panel was developed and included primer pairs targeting 178 bacteria, 96 viruses, 29 fungi, and 27 other clinically relevant pathogens commonly associated with bloodstream infections and sepsis (Supplementary Table S1). The first-round PCR amplification was performed in a 50 μL reaction volume, consisting of template DNA, primer mix, and reaction buffer from the AccuGen Pathogen Multiplex-PCR Library Prep Kit (Health SwifTech, China). The thermal cycling conditions followed the manufacturer’s protocol. After amplification, PCR products were purified using YN DNA Clean beads (included in the library prep kit) to remove primer dimers, unincorporated nucleotides, and potential contaminants.

#### 2.2.5 Library preparation for tNGS

The second-round PCR, sequencing libraries were constructed also using the AccuGen Pathogen Multiplex-PCR Library Prep Kit (Health SwifTech, China) according to the manufacturer’s instructions. The workflow included an end-repair and adapter ligation step, where amplicons were enzymatically processed to generate blunt-ended DNA fragments and ligated to indexed sequencing adapters for sample multiplexing. A subsequent indexing PCR was performed to amplify the adapter-ligated DNA fragments while incorporating unique barcodes, ensuring compatibility with downstream sequencing platforms. After amplification, the libraries underwent purification and size selection using YN DNA Clean beads to remove adapter dimers, unincorporated nucleotides, and non-specific fragments.

#### 2.2.6 Library quality control and quantification

The final libraries were then quantified using a Qubit dsDNA HS Assay Kit (Thermo Fisher Scientific, United States), and their fragment size distributions were assessed using the Agilent 2100 Bioanalyzer with the DNA 1000 Kit (Agilent Technologies, United States). Before sequencing, the libraries were normalized to equimolar concentrations, pooled, and subjected to quality control to ensure sufficient complexity and representation of the target regions.

#### 2.2.7 High-throughput sequencing and bioinformatics analysis

The enriched libraries were sequenced on an MGISEQ-2000 platform with a single-end read length of 150 bp. Quality control was conducted using FastQC (Wingett and Andrews, 2018) to ensure high-quality reads, and low-quality reads with over 10% low-quality bases or adapter contamination were discarded using Fastp (v0.23.4) (Chen et al., 2018). Clean reads were aligned to the human reference (hg38) using Bowtie2 (v2.5.4) (Langmead and Salzberg, 2012) and filtered human reads. The remaining sequences were aligned with a previously constructed reference database (containing 178 bacteria, 96 viruses, 29 fungi, and 27 other pathogens) to identify the pathogens in the sample using Burrows-Wheeler Aligner (v0.7.12) (Li and Durbin, 2009). The species-specific read number was normalized to reads per million sequencing reads (RPM). RPM ratio was the ratio between the sample and external negative control values that ran parallel to the clinical specimens throughout the workflow. The threshold criteria for determining pathogen positivity were RPM ≥ 5 and RPM ratio ≥ 10.

### 2.3 Metagenomic next-generation sequencing (mNGS) and analysis

The detailed steps are provided in Supplementary Information.

### 2.4 Spike in microbial community standard

The ZymoBIOMICS microbial community DNA standards (Zymo Research, United States) were used in this study to quantitatively evaluate the efficiency of filter membrane (Refer to Supplementary Table S2 for the Microbial List). This microbial community standard is a defined composition comprising 5 Gram-positive bacteria (*Listeria monocytogenes*, *Bacillus subtilis*, *Limosilactobacillus fermentum*, *Enterococcus faecalis*, and *Staphylococcus aureus*), 3 Gram-negative bacteria (*Pseudomonas aeruginosa*, *Escherichia coli*, and *Salmonella enterica*), and two yeast strains (*Saccharomyces cerevisiae* and *Cryptococcus neoformans*). The defined composition is well-controlled and reported by the manufacturer as the natural composition of the community.

### 2.5 Study population and sample collection

This retrospective study was conducted in the Intensive Care Unit (ICU) of Longgang District People’s Hospital of Shenzhen. We enrolled ICU patients diagnosed with sepsis who underwent pathogen detection via tNGS between August 2020 and November 2022. Additionally, blood samples from three healthy individuals were included to perform spike-in experiments using a microbial community standard for methodological validation.

The sepsis diagnostic criteria aligned with the Surviving Sepsis Campaign’s International Guidelines for Management of Sepsis and Septic Shock: 2016 (Sepsis 3.0), requiring suspected or confirmed infection along with Sequential Organ Failure Assessment (SOFA) score ≥ 2 within 24 h of ICU admission. Blood samples were collected within 24 h of sepsis onset, and additional tests were performed based on routine blood test results to assess potential secondary infections. Both regular microbial culture and tNGS methods were utilized for pathogen detection. Prior to participation, all patients provided informed oral or written consent, and the study adhered to the Declaration of Helsinki guidelines. Approval was granted by the Ethics Committee of Longgang District People’s Hospital of Shenzhen.

The blood samples extracted from the patient were divided into two groups, as follows:

Unfiltered Group: Patient blood samples were prepared within 4 h after collection (subsequently extended to 8 h) using a two-step centrifugation method. A 3 mL whole blood sample was first centrifuged at 500 *g* for 10 min at 4°C. The supernatant (plasma) was centrifuged again at 16,000 *g* for 10 min at 4°C. The final supernatant temporarily stored or transported at dry ice or −20°C.
Filtered Group: After collecting patient blood samples, filtration was conducted using a human cell-specific filtration membrane (Health SwifTech, China). The samples were subjected to centrifugation using a two-step centrifugation method. A 3 mL whole blood sample was first centrifuged at 500 *g* for 10 min at 4°C. The supernatant was centrifuged again at 16,000 *g* for 10 min at 4°C. The final supernatant was temporarily stored or transported at dry ice or −20°C before conducting the DNA/RNA extraction procedure.

## 3 Results

### 3.1 Efficacy of membrane-based selective removal of nucleated cells

**Figure 2:**
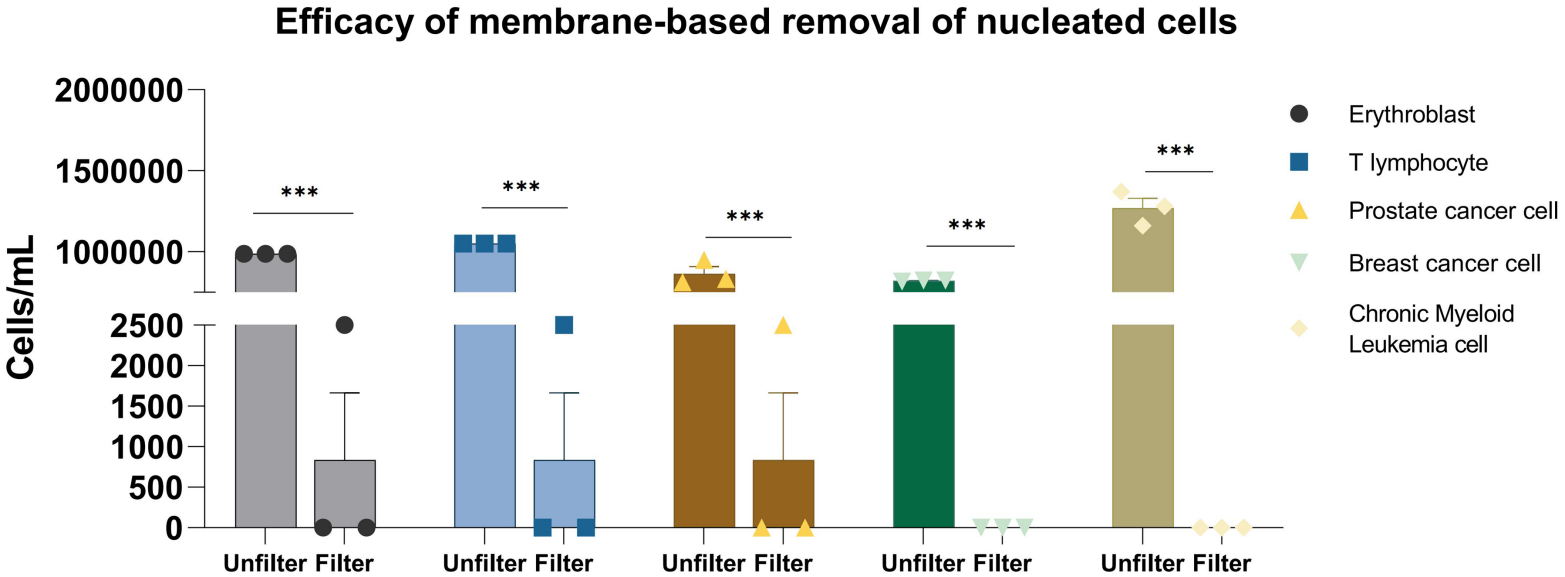
Efficacy of membrane-based removal of nucleated cells. *p < 0.05; **p < 0.01; ***p < 0.001.

To evaluate the efficacy of membrane-based specific removal of nucleated cells, we passed erythroblast, T lymphocyte, prostate cancer cell, breast cancer cell, and chronic myeloid leukemia cell through the membrane (Figure 2). Cell lines were used to ensure experimental consistency and reproducibility. These cell lines possess more stable properties, such as uniform size, morphology, and membrane characteristics, which minimize donor-to-donor variability. Furthermore, a broad range of cell types enabled a comprehensive evaluation of the filtration performance across various cell types.

The membrane filtration process demonstrated high efficacy in removing various nucleated cell types. For erythroblasts (*n* = 3), the concentration was reduced from (9.88 ± 0.00) × 10^5^ cells/mL to (8.33 ± 12.02) × 10^2^ cells/mL, achieving a reduction of approximately 99.92% ± 0.12% (*p* < 0.001, one-way ANOVA). Similarly, T lymphocyte concentration dropped from (1.05 ± 0.00) × 10^6^ cells/mL to (8.33 ± 12.02) × 10^2^ cells/mL, reflecting a 99.92% ± 0.12% reduction (*p* < 0.001). Prostate cancer cells were also effectively removed, with concentrations decreasing from (8.65 ± 6.81) × 10^5^ cells/mL to (8.33 ± 12.02) × 10^2^ cells/mL, reducing 99.90% ± 0.17% (*p* < 0.001). Filtration was fully effective for breast cancer and chronic myeloid leukemia cells, reducing their concentrations from (8.24 ± 0.46) × 10^5^ and (1.27 ± 0.11) × 10^6^ cells/mL, respectively, to undetectable levels (below 10 cells/mL, detection limit), resulting in 100% removal for both cell types (*n* = 3, SD = 0%). Overall, the membrane filtration showed strong performance across all cell types, with reduction percentages ranging from 99.90 ± 0.17 to 100% (coefficient of variation, CV < 5% for all groups).

### 3.2 Experimental validation of tNGS panels for detecting diverse and low-abundance pathogens

To evaluate the effectiveness of the primer set in capturing diversity and abundance, we used three blood samples from healthy individuals spiked with standard microbial strains in serial dilution. We then compared the results obtained from our designed pathogen primers with those from mNGS. As shown in Table 1, we compare mNGS and tNGS regarding their consistency in detecting microbial species across different DNA input amounts (10^4^ and 10^3^ copies/ml). Generally, there is a strong concordance between mNGS and tNGS in identifying dominant bacterial and fungal species, particularly at higher DNA concentrations (10^4^ copies/mL). Notably, every target assigned was effectively recognized, except for the pathogens left off the panel, such as *L. fermentum* and *B. subtilis*.

**Table 1:** Effectiveness of primer panels in capturing sequence diversity.

Input DNA amount (copy/mL)	Sample	Pathogens	mNGS (Reads/M)	tNGS (Reads/M)
10^4^	A1	*Pseudomonas aeruginosa*	1422.05	31
		*Escherichia coli*	3473.47	1,217
		*Salmonella enterica*	1537.02	341
		*Limosilactobacillus fermentum*	1213.44	–
		*Enterococcus faecalis*	1231.26	1,409
		*Staphylococcus aureus*	1158.31	103
		*Listeria monocytogenes*	1060.63	90
		*Bacillus subtilis*	16.03	–
		*Saccharomyces cerevisiae*	167.96	9
		*Cryptococcus neoformans*	220.55	45
10^3^	A2	*Pseudomonas aeruginosa*	0	4
		*Escherichia coli*	46.45	177
		*Salmonella enterica*	0.03	29
		*Limosilactobacillus fermentum*	0.03	–
		*Enterococcus faecalis*	0	158
		*Staphylococcus aureus*	0.78	9
		*Listeria monocytogenes*	0.18	11
		*Bacillus subtilis*	0	–
		*Saccharomyces cerevisiae*	0.03	1
		*Cryptococcus neoformans*	0	16
10^4^	B1	*Pseudomonas aeruginosa*	120.81	28
		*Escherichia coli*	231.66	1,178
		*Salmonella enterica*	115.22	378
		*Limosilactobacillus fermentum*	107.01	–
		*Enterococcus faecalis*	98.28	2,346
		*Staphylococcus aureus*	82.08	102
		*Listeria monocytogenes*	90.4	107
		*Bacillus subtilis*	3.5	–
		*Saccharomyces cerevisiae*	14.21	24
		*Cryptococcus neoformans*	18.12	52
10^3^	B2	*Pseudomonas aeruginosa*	54.29	5
		*Escherichia coli*	244.89	176
		*Salmonella enterica*	62.01	52
		*Limosilactobacillus fermentum*	49.88	–
		*Enterococcus faecalis*	73.38	275
		*Staphylococcus aureus*	83.89	15
		*Listeria monocytogenes*	66.72	5
		*Bacillus subtilis*	1.6	–
		*Saccharomyces cerevisiae*	10.9	15
		*Cryptococcus neoformans*	10.08	0
10^4^	C1	*Pseudomonas aeruginosa*	96.99	16
		*Escherichia coli*	1580.69	711
		*Salmonella enterica*	106.75	290
		*Limosilactobacillus fermentum*	82.67	–
		*Enterococcus faecalis*	87.29	1,345
		*Staphylococcus aureus*	72.08	81
		*Listeria monocytogenes*	67.84	36
		*Bacillus subtilis*	0.94	–
		*Saccharomyces cerevisiae*	9.24	14
		*Cryptococcus neoformans*	13.28	36
10^3^	C2	*Pseudomonas aeruginosa*	6.56	0
		*Escherichia coli*	304.27	135
		*Salmonella enterica*	4.2	32
		*Limosilactobacillus fermentum*	5.42	–
		*Enterococcus faecalis*	4.25	126
		*Staphylococcus aureus*	4.53	9
		*Listeria monocytogenes*	4.47	0
		*Bacillus subtilis*	0	–
		*Saccharomyces cerevisiae*	0.76	6
		*Cryptococcus neoformans*	1.08	3

**Figure 3:**
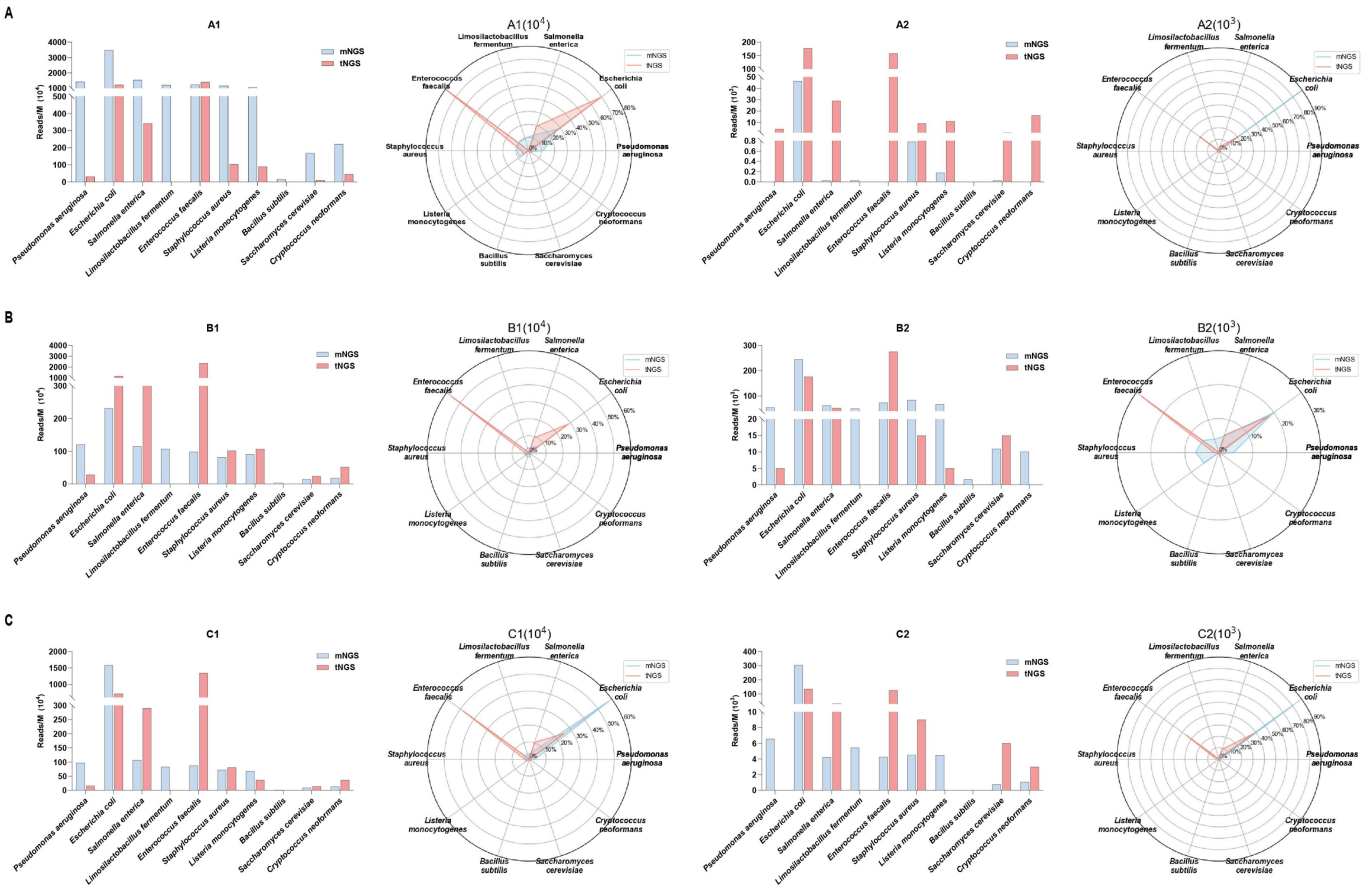
The efficacy of the primer set in capturing sequence diversity was evaluated by comparing target read numbers (Reads/M, bar chart, left) and relative abundance percentages (radar plot, right) obtained from both mNGS and tNGS analyses. These analyses were performed on three healthy individuals spiked with standard microbial strains subjected to serial dilution, corresponding to clinical specimens A **(A)**, B **(B)**, and C **(C)**. Collectively, the specimens contained 10 pathogens, including 8 bacterial and 2 fungal species.

Both methods consistently detect vital organisms such as *E. coli*, *P. aeruginosa*, *S. enterica*, and *E. faecalis* (Figure 3). At lower DNA concentrations (10^3^ copies/mL), tNGS detects a higher number of reads for low-abundance pathogens compared to mNGS, which may indicate greater sensitivity. Specifically, in sample A2, tNGS identified multiple species, including *S. enterica* (29 RPM) and *E. faecalis* (158 RPM), while mNGS results showed *S. enterica* (0.03 RPM) and *E. faecalis* (0 RPM). Donor B exhibited a higher leukocyte count compared to Donor A and C, resulting in substantially greater host DNA interference. This elevated host background disproportionately impacted tNGS at low pathogen concentrations, where primer-capture efficiency decreased compared to high-concentration B1, as human DNA competed for hybridization resources. While spike-in models inherently show donor-to-donor variability, these results still demonstrated that our tNGS panel provides more focused and specific detection, particularly for low-abundance pathogens. And our findings highlight a key methodological observation that the clinical benefits of tNGS require minimal host DNA interference.

### 3.3 Pathogen detection via tNGS following filtration of spiked standard microbial samples in whole blood

**Figure 4:**
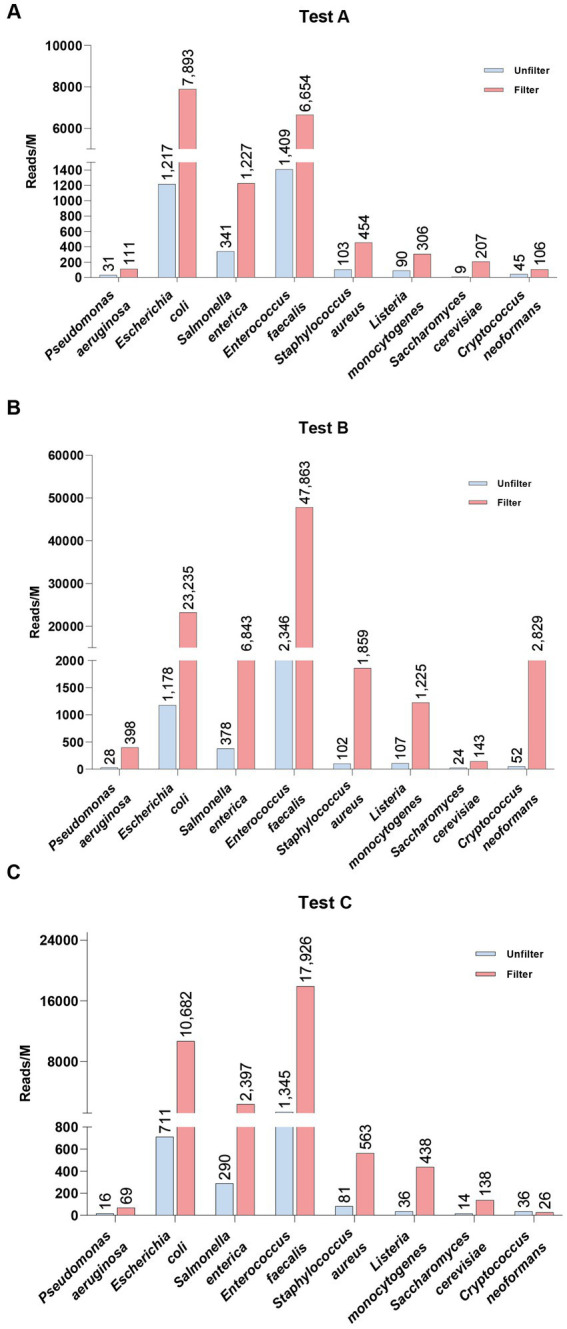
Comparison of spike-in pathogen detection in healthy whole blood samples before (Unfilter) and after filtration (Filter).

Given the noise reduction achieved through membrane-based nucleated cell removal and the effectiveness of the tNGS panel, we further combined these two methods to evaluate pathogen detection in blood samples from healthy individuals, each spiked with standard microbial strains (Figure 4). The filtration process resulted in substantial increases in pathogen reads across all samples. For example, in Test A (blood sample 1), the reads for *E. coli* rose from 1,217 to 7,893 (approximately 6.5 times higher), while *S. cerevisiae* surged from 9 to 207 reads (a 23-fold increase). In Test B (blood sample 2), *E. faecalis* exhibited a dramatic increase from 2,346 to 47,883 reads (over a 20-fold increase), and *C. neoformans* skyrocketed from 52 to 2,839 reads (about a 355-fold increase). Similarly, Test C (blood sample 3) demonstrated notable increases, with *E. coli* reaching 15 times the unfiltered count and *L. monocytogenes* increasing more than 12 times. These results underscore the significance of filtration in clinical pathogen detection, as it enhances the clarity of microbial signals.

### 3.4 Pathogen detection via tNGS following filtration of infectious clinical samples in whole blood

#### 3.4.1 Clinical characteristics of patients

In this study, we collected data from 17 infection patients, with their clinical information summarized in Supplementary Table S3. The cohort consisted of 11 males and 6 females, with a mean age of 58 years (range: 21 to 87 years). The patients presented with various infections, including bacterial pneumonia, fungal pneumonia, and pulmonary infections associated with other conditions such as myocardial infarction and cerebral hemorrhage. A total of 7 patients were diagnosed with pulmonary infections. Importantly, none of the patients had underlying viral infections. An analysis of the relationship between age, sex, and infection types revealed that older patients (particularly those aged 60 and above) were more frequently diagnosed with multiple or severe infections (Supplementary Figure S2).

#### 3.4.2 Comparison of filtration-based pathogen detection performance in blood samples from infectious clinical cases using tNGS

**Figure 5:**
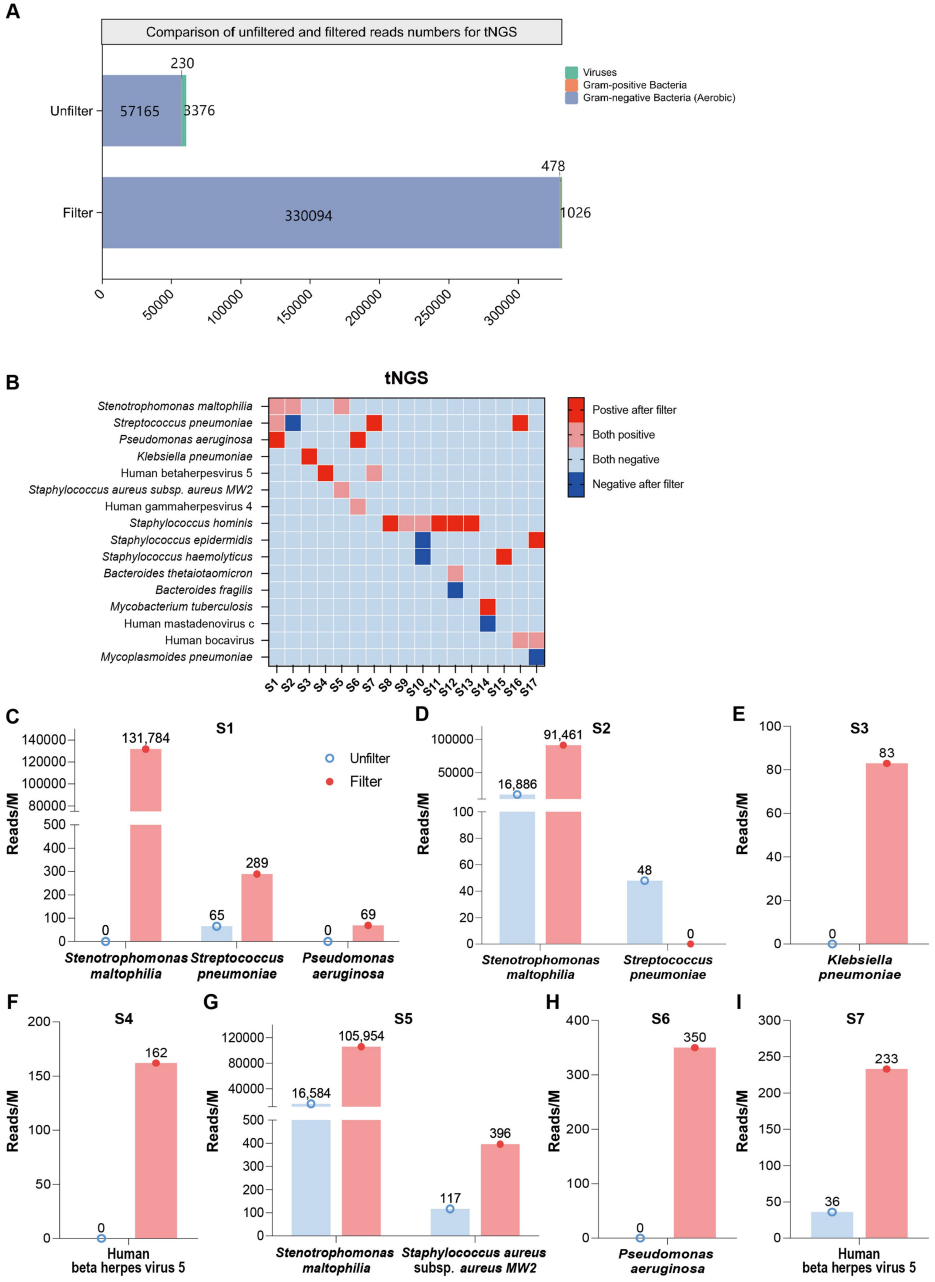
Comparison of tNGS read counts before and after filtration. **(A)** Bar graph showing the composition and enrichment of detected pathogens before and after filtration. **(B)** Heatmap showing pathogen detection in tNGS, both before and after filtration. Categories include: Positive after filter (pathogens detected post-filtration but not pre-filtration); Both positive (pathogens detected in both pre- and post-filtration samples); Both negative (pathogens detected in neither pre- nor post-filtration samples); Negative after filter (pathogens not detected pre-filtration but detected post-filtration). **(C–I)** Bar chart representing read counts across S1–S7 individual patient samples post-filtration.

To further verify the effectiveness of our combined approach, we first conducted tNGS and mNGS testing on both filtered and unfiltered samples. The tNGS results for clinical blood samples, both with and without membrane filtration, are shown in Supplementary Table S4. These results highlight the effectiveness of filtration in enhancing pathogen detection, as demonstrated by the bar chart in Figure 5A. The average read counts for gram-negative bacteria increased from approximately 57,165 before filtration to 330,094 post-filtration, while gram-positive bacteria increased from about 230 to 478 after filtration. This selective amplification of specific bacterial categories, coupled with the significant reduction of unwanted background elements, ensures that both gram-positive and gram-negative pathogens are more detectable in post-filtration samples, optimizing conditions for tNGS. The results for mNGS can be found in Supplementary Figure S3 and Supplementary Table S5. The heatmap demonstrates the specific identification of disease-causing bacteria (Figure 5B). Thirteen samples exhibited previously undetected bacteria, while five showed negative results post-filtration but still contained detectable bacteria, indicating that the filter effectively removes background noise.

The bar chart in Figures 5C–I presents a comparative analysis of read counts (reads/M) before and after filtering in S1 to S7 samples. For patient S1, in the unfiltered control group, the read counts were notably lower, with values reaching approximately 0, 65, and 0 for *Stenotrophomonas maltophilia*, *Streptococcus pneumoniae*, and *P. aeruginosa*, respectively. Post-filtration (pink bar), these counts increased substantially, rising to around 131,784, 289, and 69 (Figure 5C). Similarly, for S2, the post-filtering read counts showed a dramatic increase across all pathogens, with values rising to 91,461 for *S. maltophilia*, which represents an approximate 5.42-fold increase (Figure 5D). For patient S3, the read count for *K. pneumoniae* increased from 0 to 83 post-filtration, showing a significant improvement in detection (Figure 5E). Likewise, for patient S4, the read count for Human beta herpesvirus 5 (HHV-5) rose from 0 to 162, demonstrating a notable enhancement after filtration (Figure 5F). For patient S5, the read counts for *S. maltophilia* and *S. aureus* subsp. *aureus MW2* in the unfiltered group were 16,584 and 117, respectively. After filtration, these counts increased to 105,954 and 396, indicating an approximately 6.4-fold increase for *S. maltophilia* and a 3.4-fold increase for *S. aureus* subsp. *aureus MW2* (Figure 5G). For patient S6, the read count for *P. aeruginosa* increased from 0 to 350 (Figure 5H). For patient S7, the read count for HHV-5 rose from 36 to 233, representing a 6.5-fold increase (Figure 5I). These results highlight the substantial improvement in pathogen detection following the filtration process. Additionally, resistance genes were detected in S16 and S17 (as shown in Supplementary Table S6A), and these results were consistent with the clinical antimicrobial resistance gene testing outcomes (Supplementary Table S6B).

## 4 Discussion

This study introduced an innovative filtration membrane combined with an optimized tNGS panel for the rapid and accurate detection of pathogens in clinical blood samples. The filtration membrane selectively removed human nucleated cells, effectively enriching pathogen nucleic acids while mitigating host DNA background. This approach was particularly effective in addressing contamination caused by white blood cell lysis during sample transportation, a common issue that amplifies host-derived sequences and hampers pathogen detection (Wan Azman et al., 2019; Lippi et al., 2008).

The filtration method significantly enhanced the sensitivity of tNGS by reducing background noise, as evidenced by the enrichment of key pathogens, particularly Gram-negative bacteria and *Staphylococcus aureus*. Our results demonstrated the efficacy of filtration in increasing the read counts of specific pathogens, confirming its capacity to improve pathogen detection in complex biological samples. The selective enrichment of pathogen reads in three patient samples post-filtration further validated the filtration step’s utility in clinical diagnostics.

The tNGS panel was designed to comprehensively cover clinically relevant pathogens. This broad coverage, coupled with the filtration technology, significantly enhanced both the sensitivity and specificity of pathogen detection compared to mNGS approaches. Importantly, the tNGS panel exhibited high concordance with mNGS results while demonstrating superior performance in detecting low-abundance pathogens and resolving ambiguous cases (Sun et al., 2022; Duan et al., 2021; Hong et al., 2023). This consistency and accuracy highlight the potential of tNGS as a robust diagnostic tool in clinical microbiology. Our approach facilitated a detailed genetic analysis of pathogens, providing a comprehensive understanding of the microbial landscape in clinical samples. This synergistic workflow of wet and dry lab research marked a departure from conventional methods, aligning with the growing trend of high-throughput sequencing in clinical diagnostics (Kato et al., 2023; Murphy et al., 2023). The enhanced sensitivity and specificity of pathogen detection provided by this method contribute to a more refined understanding of sepsis etiology, potentially leading to earlier and more accurate diagnoses.

Moreover, previous methods employing a straightforward syringe-based filtration system allow for the retention of viable bacteria on a filter paper, while effectively washing out lysed blood cells, thereby reducing interference in subsequent analyses (Narayana Iyengar et al., 2021). However, this method presents several challenges, including incomplete bacterial capture, risk of filter clogging, selective retention of bacteria, reduced bacterial viability, labor intensiveness, and variability in filter paper quality that impacts reproducibility.

The primary limitation of this study lies in the restricted diversity of clinical samples, which affects the generalizability and robustness of our findings. A diverse sample set is essential to reflect the full spectrum of pathogens encountered in clinical settings; however, due to limitations in sample availability, our analysis included only a limited number of clinical specimens. This restriction may hinder the broad applicability of our conclusions, as our results might not fully capture the variability and prevalence of pathogens across different clinical scenarios. The constrained sample diversity could also affect the evaluation of our novel filtration device and sequencing methods when applied to various pathogen types and infection contexts. Additionally, another potential limitation is the selective amplification bias during the filtration process. Specifically, Gram-negative bacteria exhibited a more significant increase in read counts compared to Gram-positive bacteria after filtration. This selective amplification can be attributed to the structural differences between these two groups of bacteria. Gram-negative bacteria, with their outer membrane composed of lipopolysaccharides (LPS), interact differently with the filtration membrane compared to Gram-positive bacteria, which possess a thicker peptidoglycan layer (Gong et al., 2023; Bishop et al., 2000). The outer membrane of Gram-negative bacteria may weaken their interaction with the filtration membrane, allowing them to be less retained, whereas the thicker cell wall of Gram-positive bacteria leads to a stronger interaction (Pasquina-Lemonche et al., 2020), possibly causing Gram-negative bacteria to be more readily enriched in the post-filtration sample. While Gram-negative bacteria exhibited higher amplification in terms of fold increase in read counts, it is important to note that Gram-positive bacteria also showed significant amplification, contributing to the overall sensitivity of pathogen detection. Gram-positive bacteria, despite the relatively lower amplification in comparison to Gram-negative bacteria, still play a crucial role in the overall detection of pathogens post-filtration.

Since blood samples undergo filtration immediately after collection, host cells, including leukocytes harboring intracellular pathogens, are largely removed before nucleic acid extraction. This may reduce the detection of intracellular pathogens which persist within host cells. Moreover, certain intracellular pathogens, such as *Plasmodium* spp. and *Trypanosoma* spp., have extracellular phases that allow their detection even in the absence of leukocytes. Similarly, *Brucella* spp. and *L. monocytogenes* exist in both intracellular and extracellular states, ensuring that a fraction of their DNA remains detectable in filtered plasma (Baldwin and Goenka, 2006). Our assessment is that the advantages of leukocyte depletion in eliminating host DNA contamination, increasing sequencing sensitivity, and improving pathogen signal detection outweigh the drawbacks of potentially missing some persistent pathogens.

Finally, another critical consideration in NGS-based pathogen detection is that detecting microbial nucleic acids does not inherently confirm pathogenicity. NGS technology can identify both viable and non-viable microbial fragments, environmental contaminants and commensal organisms that may be harmless components of the normal microbiome (Dulanto Chiang and Dekker, 2019). In our study, the observed discrepancies between tNGS and conventional blood culture reflect this paradigm: blood culture’s requirement for viable, cultivable pathogens led to a 65% underdetection rate compared to tNGS, primarily due to (1) restrictive growth conditions (e.g., anaerobic Fusobacterium in aerobic media), (2) slow-growing pathogens exceeding standard incubation timelines (e.g., Mycobacterium) and (3) antimicrobial pretreatment suppressing microbial viability. To address specificity concerns inherent to NGS, tNGS employed primer-directed enrichment targeting clinically actionable pathogens, achieving higher DNA recovery than culture while excluding non-viable background microbes through stringent bioinformatics filters. Although mNGS reported broader diversity, most of its additional taxa were classified as environmental contaminants (e.g., reagent-derived Pseudomonas), reinforcing tNGS’s clinical utility over exhaustive microbial cataloging. These complexities underscore the need for contextual interpretation of sequencing data, as mere sequence detection may not indicate active infection. Integrating NGS with host immune response profiling, culture-based validation and clinical assessment will be essential to improve diagnostic accuracy and reduce false-positive identifications.

Future research should prioritize expanding clinical sample diversity to enhance generalizability and develop methods to distinguish infectious agents from non-pathogenic or residual DNA. Integrating NGS with advanced bioinformatic filters, optimized sample preparation, and complementary diagnostic assays could increase sequencing data’s specificity and diagnostic value, positioning NGS as a more definitive tool for clinical pathogen identification. This method also promises to improve early cancer detection techniques that support genetic and prenatal health.

## 5 Conclusion

In this study, we demonstrated the effectiveness of a novel filtration-based approach combined with tNGS for the rapid and accurate detection of pathogens in whole blood samples. The filtration step significantly enhanced the sensitivity and specificity of pathogen detection by selectively enriching microbial nucleic acids while reducing host-derived background noise. The successful amplification of pathogen signals across patient samples highlights the potential of this approach to improve clinical diagnostics, particularly in complex biological samples where conventional methods fall short. Future research involving a more extensive and diverse sample set will be crucial to further validate the utility of this method and expand its applicability across a broader range of infectious diseases. This filtration-enhanced tNGS method represents a promising advancement in pathogen detection, offering a more reliable and efficient tool for real-world clinical diagnostics.

## Data Availability

The datasets generated and analyzed in this study are available in online repositories: https://www.ncbi.nlm.nih.gov/. The tNGS data can be found at NCBI under accession number PRJNA1222127, and the mNGS data can be found at NCBI under accession number PRJNA1224417.
