# Supplementary material for: Enhancing bloodstream infection diagnostics: a novel filtration and targeted next-generation sequencing approach for precise pathogen identification
Source: Front Microbiol. 2025 Mar 20;16:1538265. doi: 10.3389/fmicb.2025.1538265 (PMC11965694; doi:10.3389/fmicb.2025.1538265)
Supplement: Supplementary file 1 [file Data_Sheet_1.docx]

**Supplementary information**

Enhancing bloodstream infection diagnostics: A novel filtration and targeted next-generation sequencing approach for precise pathogen identification

Ting-Syuan Lin^1,2,3,†^, Zihao Zhu^1,2,3,†^, Xiaohong Lin^4^, Hsi-Yuan Huang^1,2,3^, Liping Li^1,2,3^, Jing Li^1,2,3^, Jie Ni^1,2,3^, Peizhi Li^5^, Lanchun Chen^4^, Weixin Tang^4^, Huixin Liu^4^, Xiaolong Se^4^, Mingfei Xie^4^, Canling Long^6^, Chih-Min Chiu^7^, Szu-Han Fang^7^, Jiaming Zhao^7^, Yang-Chi-Dung Lin^1,2,3,*^, Xuetao Yu^4,*^, Hsien-Da Huang^1,2,3,8,*^

*^1^School of Medicine, The Chinese University of Hong Kong, Shenzhen, Shenzhen, China*

*^2^Warshel Institute for Computational Biology, School of Medicine, The Chinese University of Hong Kong, Shenzhen, Shenzhen, China*

*^3^**Guangdong Provincial Key Laboratory of Digital Biology and Drug Development, The Chinese University of Hong Kong, Shenzhen, Shenzhen, China,*

*^4^Department of Critical Care Medicine, The Second Affiliated Hospital, School of Medicine, The Chinese University of Hong Kong, Shenzhen & Longgang District People's Hospital of Shenzhen, Shenzhen, China*

*^5^Shanya life-tech Co. Ltd, Guangzhou, China*

*^6^Central Laboratory, The Second Affiliated Hospital, The Chinese University of Hong Kong, Shenzhen & Longgang District People's Hospital of Shenzhen, Shenzhen, China*

*^7^Health SwifTech Co. Ltd., Shenzhen, China*

*^8^Department of Endocrinology, Key Laboratory of Endocrinology of National Health Commission, Peking Union Medical College Hospital, Chinese Academy of Medical Sciences & Peking Union Medical College, Beijing, China*

† These authors contributed equally to this work as first authors.

* Correspondence: Yang-Chi-Dung Lin (yangchidung @cuhk.edu.cn); Xuetao Yu ([yxt1066@sina.cn](mailto:yxt1066@sina.cn)); Hsien-Da Huang (huanghsienda@cuhk.edu.cn).

**Figure S1. Macromolecule of leukosorb membranes.**

**Figure S2. Age distribution of patients by gender and age group.**

**Figure S3. Bar graph showing the composition and enrichment of detected pathogens before and after filtration in mNGS.**

1. **Materials and Methods**

**1.1** **Composition of nucleated cell-capturing substrates**

The distinctive feature of the substrate capable of capturing nucleated cells lies in its composition, which includes materials such as leukosorb membranes, triacetate cellulose, acetate cellulose, glass cellulose, quartz cellulose, nitrate cellulose, regenerated cellulose, or nylon-based substrates (Figure S1).


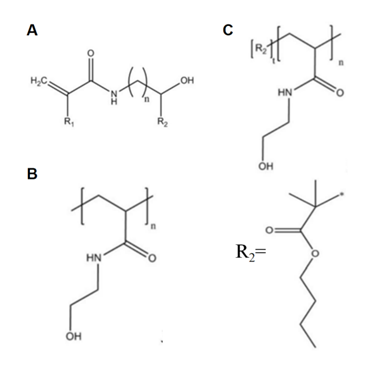


Figure S1. Macromolecule (A) In the chemical structure, R1 is independently selected from hydrogen, methyl, ethyl, hydroxy, C1 to C12 alkyl, and phenyl. R2 is independently selected from hydrogen, methyl, ethyl, C1 to C6 alkyl, amino, and phenyl. The integer n ranges from 1 to 5. (B) n is an integer ranging from 10 to 50. (C)t is an integer ranging from 50 to 90, and n is an integer ranging from 10 to 50.

**1.2 Metagenomic next-generation sequencing (mNGS)**

**1.2.1 DNA extraction**

Total DNA and RNA were co-extracted from the samples using the AccuGen Nucleic Acid Extraction (DNA/RNA) Kit (Health SwifTech, China) following the manufacturer's protocol.

**1.2.2 Library construction**

The construction of DNA libraries is a critical step in next-generation sequencing (NGS) applications. To ensure the reproducibility and accuracy of experimental results, a manufacturer's protocol for DNA library construction is necessary (AccuGen Pathogen metagenomic Library Prep Kit, Health SwifTech, China). The Standard Operating Procedure (SOP) described in this study provides a detailed and organized protocol for DNA library construction using MGI-DNA technology. The SOP involves several specific steps, including DNA fragmentation using a Covaris sonicator, end-repair, A-tailing, ligation of DNA index X adapters, PCR amplification, and purification using DNA Clean Beads. The use of unique barcodes in the DNA index adapters allows for multiplexed sequencing, which increases the efficiency and throughput of the sequencing process. The SOP ensures the purity and quality of the DNA libraries, which is critical for downstream applications such as variant calling and gene expression analysis. The SOP also includes detailed instructions for equipment and reagent preparation, as well as troubleshooting tips for common issues that may arise during the library construction process. Overall, this SOP provides a reliable and efficient method for DNA library construction that can be used in a variety of research applications. The use of this SOP will facilitate the standardization of DNA library construction and improve the reproducibility and accuracy of NGS experiments.

The DNA libraries were combined in equal amounts and the resulting sequencing library pool was assessed for concentration using the Qubit fluorometer manufactured by Thermo Fisher. Subsequently, MGI sequencing was carried out on either a MGISEQ-2000 platform, which had the capacity to process up to five samples per run, with a 2×150-nt paired-end read configuration following the manufacturer's protocol.

**1.2.3 mNGS bioinformatics analysis**

The mNGS bioinformatics analysis process is as follows:

(1) Pre-processing of second-generation sequencing reads involves the removal of adapters and low-quality, low-complexity sequences. Sample data volume, Q30 scores, and other quality control metrics are computed.

(2) Human reference genomes (hg38, hg19) and the human mitochondrial reference genome are selected as host reference genomes. The alignment software, bowtie2, is chosen to construct the alignment database. After alignment, human-derived sequences are filtered out.

(3) Relevant bacterial, fungal, parasitic, and viral reference genomes are selected from NCBI RefSeq, GenBank, and the Reference Viral Database (RVDB). Utilizing NCBI taxonomy classification and evolutionary relationships, a pathogen database corresponding to taxonomy trees is constructed using the kraken2 software.

(4) The processed non-human-derived sequencing sequences are cut into k-mers using kraken2. These k-mers are aligned to the aforementioned pathogen database to obtain their Lowest Common Ancestor (LCA) taxonomy and alignment counts. The data is utilized to construct a classification tree, calculating the cumulative weights for each root-to-leaf path. The maximum weight corresponds to the classification tree of the sequence, yielding the annotation results for each sequence. The bracken software is employed for species sequence count correction.

(5) The results are organized and subjected to statistical analysis, including metrics such as species-specific sequence counts, species-corrected sequence counts, RPM, and relative abundances, providing technical interpretations of the findings.

1. **Results**
   1. **Clinical characteristics of patients**

An analysis of the relationship between age, sex, and infection types revealed that older patients (particularly those aged 60 and above) were more frequently diagnosed with multiple or severe infections (Figure S2).


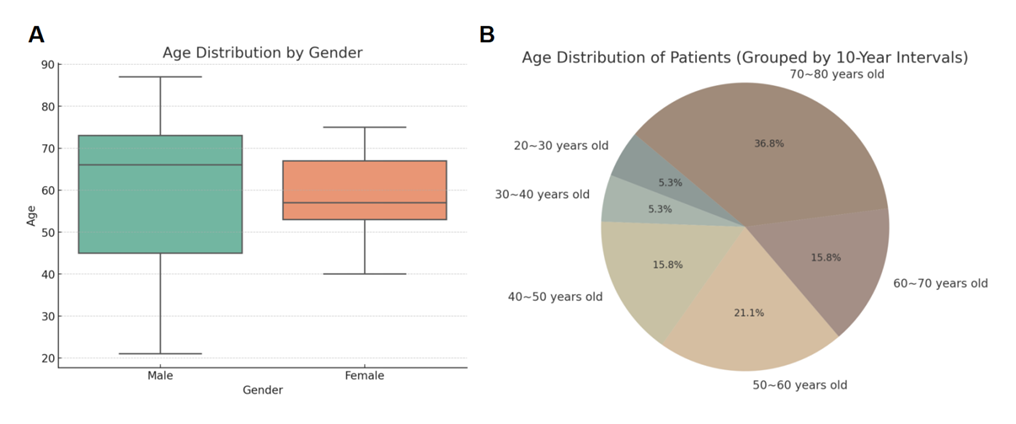


**Figure S2.** Age distribution of patients by gender and age group. (A) The boxplot illustrates the age distribution between male and female patients. Males (n = 11) exhibit a wider range of ages compared to females (n = 6), with a median age slightly higher in the male group. The interquartile range and overall distribution highlight that older patients are more common in both gender groups, but males have a broader variability in age. (B) The pie chart categorizes patients into 10-year age intervals, showing that the majority of patients (36.8%) fall in the 70–80 years old range. Other significant age groups include 50–60 years (21.1%) and 60–70 years (15.8%), reflecting a predominance of older individuals within the study cohort.

- 1. **Pathogen detection via tNGS following filtration of infectious clinical samples in whole blood**


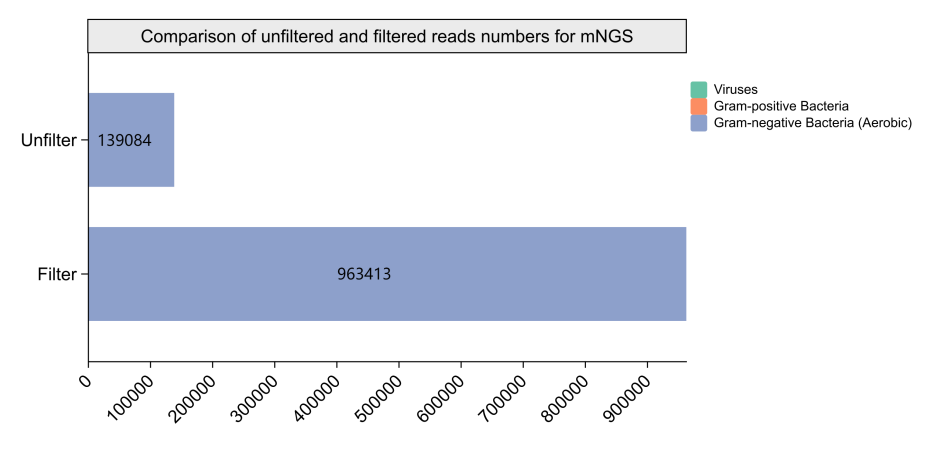


**Figure S3.** Bar graph showing the composition and enrichment of detected pathogens before and after filtration in mNGS.
